# Use of Private Sector Workforce Respiratory Disease Short-Term Disability Claims to Assess SARS-CoV-2, Mexico, 2020

**DOI:** 10.3201/eid2801.211357

**Published:** 2022-01

**Authors:** Mauricio Hernandez-Avila, Marcela Tamayo-Ortiz, Waldo Vieyra-Romero, Hector Gutierrez-Diaz, Rodrigo Zepeda-Tello, David Barros-Sierra, Rebeca Velasco-Reyna, Emmanuell Ramirez-Polanco, Manuel Ortega-Alvarez

**Affiliations:** Mexican Social Security Institute, Mexico City, Mexico

**Keywords:** coronavirus disease, COVID-19, private sector, workforce, disability claims, healthcare, severe acute respiratory syndrome coronavirus, SARS-CoV-2, coronaviruses, viruses, respiratory infections, syndromic surveillance, private sector, workforce, occupational health, zoonoses, Mexico

## Abstract

We examined respiratory disease short-term disability claims submitted to the Mexican Social Security Institute during 2020. A total of 1,631,587 claims were submitted by 19.1 million insured workers. Cumulative incidence (8.5%) was 3.6 times higher than that for January 2015‒December-2019. Workers in healthcare, social assistance, self-service, and retail stores were disproportionately affected.

During February 28–December 31, 2020, Mexico officially reported 1.8 million coronavirus disease (COVID-19) cases and 326,612 all-cause excess deaths (representing a 45.1% excess of total expected deaths for the time period) (*1*). Surveillance in Mexico is based on sentinel sampling capturing a systematic subset of COVID-19 cases, with limited access to widespread testing (*2*). Despite concern for health outcomes among healthcare workers, studies examining the effects of COVID-19 across different occupational groups are lacking. To describe the severe acute respiratory syndrome coronavirus 2 (SARS-CoV-2) epidemic among private-sector workers, we analyzed respiratory disease short-term disability claims (RD-STDC) submitted during 2020 to the Mexican Social Security Institute (Spanish acronym IMSS) by 19.1 million workers insured by the IMSS.

## The Study

We extracted workers’ social security number, age, sex, start date, duration of temporary disability, and clinical diagnosis (International Classification of Diseases, 10th Revision‒, codes for COVID-19 [U070, U071, U072, U07E, U07S, B342, and B972], acute respiratory diseases [J01, J04-J06, J20, and J21], influenza [(J10, J11], pneumonia [J12‒J18], and other [J029, J00X, J02X, J039, and J22X]) from the IMSS STDC database for 2015─2020. We then linked workers through their social security number to their employer’s activity, sector, and subsector and to IMSS registries for SARS-CoV-2 testing information (real-time PCR).

We described RD-STDC by using weekly rates per 10,000 workers and plotted the claims by epidemiologic week and by economic sector and subsector. We use industry sectors (subsectors) included in the North American Industry Classification System (NAICS) codes: agriculture, forestry, fishing, and hunting (NAICS-Sector 11); mining, quarrying, and oil and gas extraction (NAICS-Sector 21); manufacturing (NAICS-Sectors 31–33), food manufacturing, textile mills, transportation equipment, computer and electronic products; construction (NAICS-Sector 23), retail trade (NAICS-Sectors 44 and 45), food and beverage stores, general merchandise stores, self-service and retail stores); communications and transportation (NAICS-Sectors 48 and 49); services for companies, homes, and people (NAICS-Sectors 56 and 72, accommodation, food services, and drinking places); and social and community services (NAICS-Sectors 61, 62, and 71, educational services, arts, entertainment, and recreation, health care and social assistance).

We evaluated RD-STDC outbreak severity by using the moving epidemic method (*3*) to estimate epidemic threshold and epidemic intensity (EI) level (i.e., low, medium, high, and very high) for the 2015–2019 winter seasons. We defined the epidemic threshold by the 1-sided 95% CI around the arithmetic mean of the 30 highest preepidemic/postepidemic weekly rates and the EI (low, medium, high and very high intensity) by using the 1-sided 95% CI around the geometric mean of 30 highest weekly epidemic rates. We set CI limits at 40%, 90%, and 97.5%.

We estimated industry-, sector- and subsector-specific cumulative RD-STDC incidence by dividing the sum of RD-STDC by the worker population registered at IMSS for each year and compared 2015–2019 (baseline) with 2020 by using risk ratios. Cumulative COVID-19 incidence was calculated by applying the percentage of workers with a positive SARS-CoV-2 test with the RD-STDC weekly number and dividing the cumulative sum of COVID-19 cases (n = 1,029,122) by the worker population. We calculated lost workdays (LWDs) by adding all authorized days and excess LWDs as the observed minus the expected number of LWDs in weeks above the threshold (90th percentile) for each week. We calculated proportions of LWD rates by dividing the number of workdays lost to RD-STDC per year by the total number of available workdays. This project was deemed a public health surveillance and did not require institutional review board approval. We used Stata version 15.1 (https://www.stata.com) for all graphical and statistical analyses.

**Figure 1:**
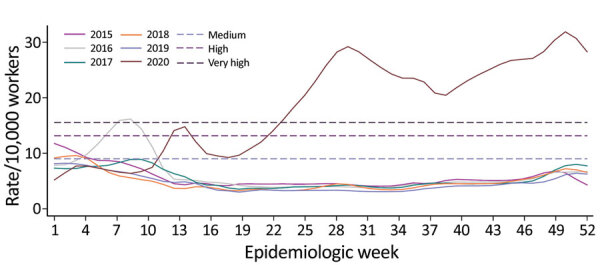
Annual incidence rates of work absenteeism related to respiratory diseases short-term disability claims per 10,000 workers for workers insured by the Mexican Social Security Institute, by epidemiologic week, Mexico, 2015–2020. Smoothed series was determined by using locally weighted scatterplot s*moothing*. Epidemic threshold was estimated by using the moving epidemic method and observed values for 2015‒2019. Dashed lines indicate epidemic thresholds and epidemic intensities (i.e., low, medium, high, and very high) for the 2015–2019 winter seasons.

**Figure 2:**
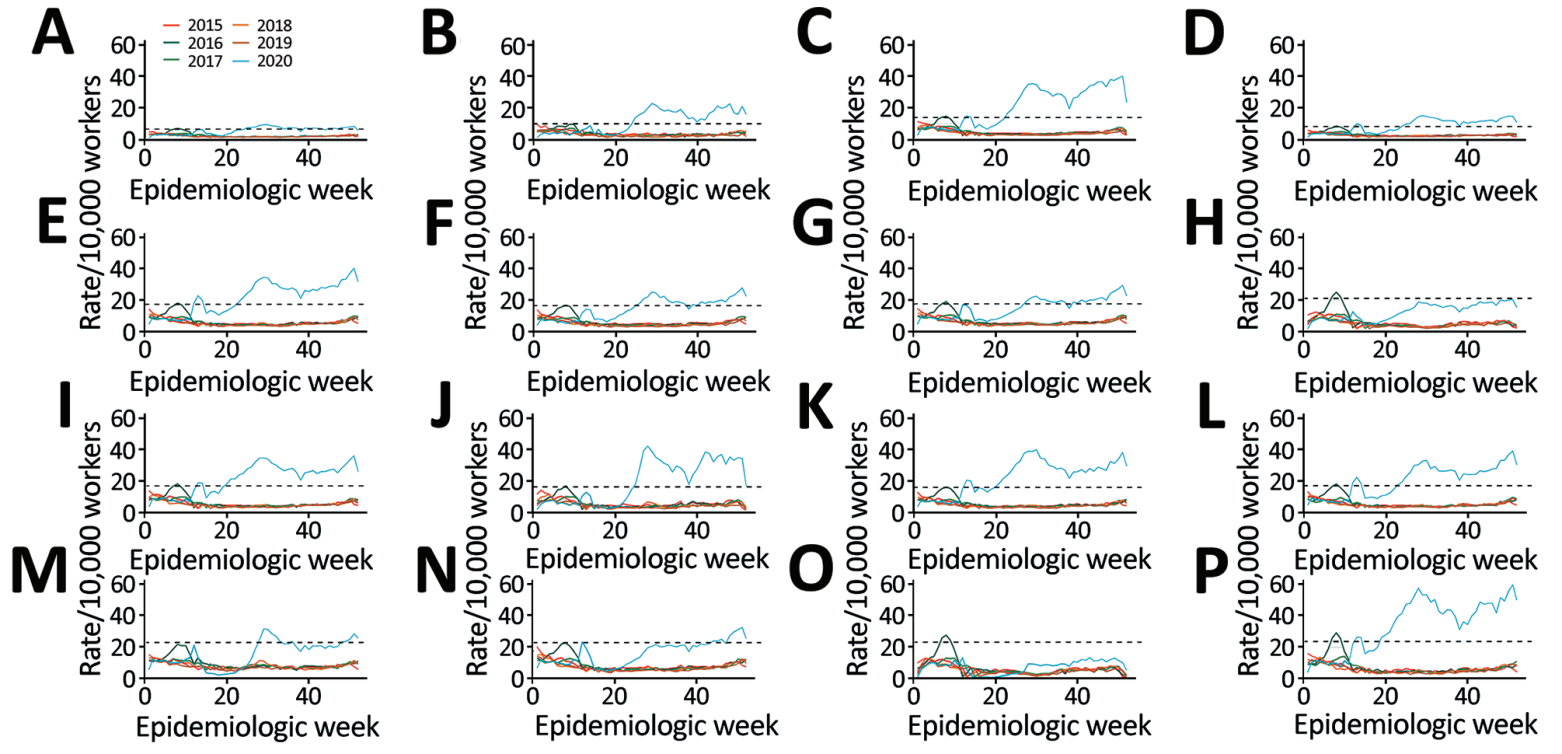
Annual incidence rate of work absenteeism related to respiratory diseases short-term disability claims per 10,000 workers for workers in selected industry sectors (A‒H) and subsectors (I‒P) insured by the Mexican Social Security Institute, by epidemiologic week, Mexico, 2015–2020. Smoothed series was determined by using locally weighted scatterplot s*moothing*. Black dashed lines indicate epidemic thresholds, estimated by using the moving epidemic method and observed values for 2015‒2019. A) Agriculture, forestry, fishing and hunting; B) mining, quarrying, and oil and gas extraction; C) manufacturing; D) food manufacturing; E) transportation equipment; F) construction; G) retail trade; H) food and beverage stores; I) self-service and retail stores; J) communications and transportation; K) services for companies, homes and people; L) accommodation; M) food services and drinking places; N) social and community services; O) educational services, arts, entertainment, and recreation; P) health care and social assistance.

Among 19.1 million insured workers (mean age 36.9 years, 61.5% males), we identified 1,631,587 workers with an RD-STDC (53.4% males, mean age 35.1 years, interquartile range 26.0–43.3 years). Weekly RD-STDC rates increased sharply (Figure 1) and reached a medium EI (first peak 35,707 RD-STDC reported); claims decreased but remained above medium EI level until May, when the slope increased to a maximum in week 29 (July 18, second peak 62,542 RD-STDC reported), crossing into the EI level of very high intensity. From this date, RD-STDC frequency decreased but remained 5‒6 times higher than historical values until week 38 (September 13), when RD-STDC increased, reaching a third peak (71,785 RD-STDC reported) during week 51 (December 20). Up to week 53, a total of 1,543,600 cumulative RD-STDC were reported, with an estimated cumulative incidence of 8.0%. The EI of epidemic curves varied by industry sector and subsector (Figure 2).

A total of 20% of workers who had an RD-STDC were tested for SARS-CoV-2; 59% were positive. The estimated number of symptomatic COVID-19 cases among IMSS-affiliated workers was 910,073 (cumulative incidence 5%). The median RD-STDC granted was 10 days (interquartile range 4–14 days), representing an excess of 17.4 million workdays lost compared with that during 2015‒2019.

We compiled the cumulative RD-STDC incidence for different industry sectors (Table), which ranged from 3% for agriculture, forestry, fishing, and hunting to 10% for retail trade and manufacturing. When we compared these data with those for 2015–2019, we found that the total RD-STDC during 2020 were 2.9‒5.0 times higher in all sectors. The amounts of total workdays lost (WDL) attributed to RD-STDC (Appendix Table) were highest among the manufacturing sector (0.44%; WDL 6.10 million). Among subsectors, WDL ranged from 0.14% (WDL 0.24 million) for educational services, arts, entertainment, and recreation to 0.80% (WDL 0.64 million) for health care and social assistance. 

**Table:** Characteristics for 19 million insured workers by industry sector and selected subsector, from the Mexican Social Security Institute, Mexico, January 1‒December 30, 2020*

Economic activity of employer	No. insured workers†	No. workers claiming an RD-STDC	Cumulative RD-STDC incidence, %	RR (95% CI)	PCR-positive results, %	COVID-19 attack rate, %	Estimated no. COVID-19 cases	Work days lost, millions	Work days lost, %
Agriculture, forestry, fishing and hunting	757,997	22,461	3.0	2.9 (2.9‒3.0)	55.9	1.5	11,638	0.21	0.11
Mining, quarrying, and oil and gas extraction	119,946	7,360	6.1	4.3 (4.2‒ 4.5)	66.0	3.9	4,645	0.09	0.28
Manufacturing	5,438,831	558,057	10.3	5.0 (5.0‒ 5.0)	60.5	5.9	322,786	6.10	0.44
Food manufacturing	920,558	96,000	10.4	3.9 (3.8‒ 3.9)	58.7	5.8	53,373	1.00	0.43
Textile mills	281,521	30,625	10.9	4.3 (4.3‒4.4)	60.3	6.3	17,653	0.32	0.42
Transportation equipment	1,062,508	122,659	11.5	6.0 (6.0‒6.1)	59.0	6.5	69,563	1.34	0.50
Computer and electronic products	64,837	4,235	6.5	4.8 (4.5‒5.0)	64.0	4.0	2,566	0.05	0.29
Construction	1,487,563	70,416	4.7	3.7 (3.6‒3.7)	61.6	2.7	40,893	0.74	0.19
Retail trade	4,040,863	421,940	10.4	3.9 (3.9‒3.9)	56.3	5.6	225,901	4.42	0.43
Food and beverage stores	825,597	90,864	11.0	4.4 (4.3‒4.4)	57.9	6.1	50,311	0.98	0.46
General merchandise stores	798,679	85,065	10.7	4.0 (3.9‒4.0)	58.1	5.9	46,849	0.89	0.43
Self-service and retail stores	844,805	139,032	16.5	3.6 (3.6‒3.7)	49.3	7.7	65,302	1.41	0.66
Communications and transportation	1,213,211	88,593	7.3	3.1 (3.0‒3.1)	62.4	4.3	51,898	1.00	0.32
Services for companies, homes, and people	4,399,135	356,411	8.1	2.8 (2.8‒2.8)	58.4	4.4	191,968	3.63	0.31
Accommodation	323,789	32,751	10.1	2.4 (2.4‒2.5)	52.9	4.8	15,573	0.31	0.37
Food services and drinking places	564,531	57,576	10.2	2.7 (2.7‒2.7)	58.8	5.4	30,760	0.55	0.35
Social and community services	1,655,074	106,349	6.4	2.5 (2.5‒2.6)	61.8	3.6	60,345	1.21	0.28
Educational services, arts, entertainment, and recreation	591,471	26,447	4.5	1.4 (1.4‒1.5)	57.3	2.1	12,635	0.24	0.14
Health care and social assistance	318,621	49,768	15.6	5.5 (5.4‒5.6)	63.0	9.5	30,208	0.64	0.80
General	19,112,620	1,631,587	8.5	3.6 (3.6‒3.7)	59.2	4.8	910,073	17.4	0.35

*There were 5,272 workers who lacked information on economic activity. Workers from public sectors were excluded. RR estimated comparing 2020 with baseline period of 2015–2019. COVID-19, coronavirus disease; RT-STDC, respiratory disease short-term disability claims RR, relative risk. †As of February 2020.

## Conclusions

Workers in multiple industry sectors and subsectors were disproportionately affected by the SARS-CoV-2 pandemic. The above-average cumulative incidence of RD-STDC among some subsectors suggests an occupational link to infection. Healthcare workers had a high cumulative RD-STDC incidence (15.6%). This incidence was similar for persons who worked near or frequently with other workers or who have frequent customer contact but are not exposed to SARS-CoV2 directly, such as workers in manufacturing, food, and retail. In contrast, lower attack rates were observed within the educational services, arts, entertainment, and recreation subsectors. In Mexico, after their closure on March 16, 2020, schools continued with distance learning activities until August 30, 2021, when in-person instruction was reestablished.

Real-time analysis of RD-STDC by sector and subsector enables detection of outbreaks and rapid implementation of mitigation measures such as contact tracing, reinforcing hand hygiene, universal masking, and social distancing. RD-STDC rates might also be used to guide vaccination priorities aimed at protecting workers at higher risk for infection.

Our study’s first limitation is that COVID-19 cases among workers might have been underestimated because not all symptomatic workers requested RD-STDC. In a recent serosurvey of IMSS insured workers, 26%‒40% of workers with SARS-CoV-2 antibodies and symptoms compatible with COVID-19 sought RD-STDC (D. Barros-Sierra Cordero et al., IMSS, pers. comm., 2021 Jan 10), suggesting that real symptomatic COVID-19 attack rates could be 3‒4 times higher. Second, asymptomatic or minimally symptomatic workers infected with SARS-CoV-2 are not reflected in this analysis. Third, some RD-STDCs might have been related to diseases other than COVID-19. Fourth, not all workers included in our denominator sought care within IMSS facilities, leading to incomplete capture of RD-STDC and a potential underestimation of the magnitude of the epidemic. Fifth, we did not examine geographic differences in RD-STDC claims. It is possible that economic sectors and subsectors less affected were concentrated in areas of low epidemic intensity in the country, resulting in lower RD-STDC rates, which could account for the low rates observed in agriculture, forestry, fishing, and hunting.

Predictive models based on the RD-STDC surveillance system are currently used by the IMSS to alert large manufacturing sites and other businesses of possible workplace outbreaks. Information provided by this system complements other surveillance systems for monitoring epidemics and inform decision-making by health authorities. Our data highlight the need for including respiratory disease claims in pandemic preparedness planning.

Appendix Additional information on use of private sector workforce respiratory disease short-term disability claims to assess SARS-CoV-2, Mexico, 2020.
