# Supplementary material for: Use of Private Sector Workforce Respiratory Disease Short-Term Disability Claims to Assess SARS-CoV-2, Mexico, 2020
Source: Emerg Infect Dis. 2022 Jan;28(1):214–8. doi: 10.3201/eid2801.211357 (PMC8714224; doi:10.3201/eid2801.211357)
Supplement: Appendix — Additional information on use of private sector workforce respiratory disease short-term disability claims to assess SARS-CoV-2, Mexico, 2020. [file 21-1357-Techapp-s1.pdf]

Article DOI: https://doi.org/10.3201/eid2801.211537

Use of Private Sector Workforce Respiratory Disease Short-Term Disability Claims to Assess SARS-CoV-2, Mexico, 2020

Appendix

Appendix Table. Workdays lost for 20 million IMSS-insured workers by sector and selected subsector of economic activity, Mexico, January 1–December 30, 2020*

Economic activity of employer	Mean (SD)	Median (interquartile range)
Agriculture, Forestry, Fishing and Hunting	9.4 (11.2)	7 (3–14)
Mining, Quarrying, and Oil and Gas Extraction	11.9 (12.5)	11 (5–14)
Manufacturing	10.9 (9.7)	10 (4–14)
Food Manufacturing	10.4 (10.1)	10 (3–14)
Textile Mills	10.5 (9.1)	10 (4–14)
Transportation Equipment	10.9 (9.2)	10 (5–14)
Computer and Electronic Products	11.2 (9.5)	11 (3–14)
Construction	10.5 (11.1)	10 (3–14)
Retail Trade	10.5 (9.4)	10 (4–14)
Food and Beverage Stores	10.8 (9.5)	10 (4–14)
General Merchandise Stores	10.5 (9.3)	10 (4, 14)
Self-service and retail stores	10.2 (8.8)	9 (3–14)
Communications and transportation	11.3 (11.9)	10 (4–14)
Services for companies, homes and people	10.2 (9.9)	9 (3–14)
Accommodation	9.5 (8.8)	8 (3–14)
Food Services and Drinking Places	9.6 (9.3)	8 (3–14)
Social and community services	11.4 (10.7)	11 (5–14)
Educational Services, Arts, Entertainment, and Recreation	9.2 (11.0)	7 (3–14)
Health Care and Social Assistance	12.8 (9.9)	13 (7–14)
General	10.7 (10.0)	10 (4–14)

*IMSS, Mexican Social Security Institute (Spanish acronym IMSS).
